# Regulatory and effector B cell cytokine production in patients with relapsing granulomatosis with polyangiitis

**DOI:** 10.1186/s13075-016-0978-1

**Published:** 2016-04-04

**Authors:** Judith Land, Wayel H. Abdulahad, Jan-Stephan F. Sanders, Coen A. Stegeman, Peter Heeringa, Abraham Rutgers

**Affiliations:** Department of Rheumatology and Clinical Immunology, AA21, University of Groningen, University Medical Center Groningen, Hanzeplein 1, 9713 GZ Groningen, The Netherlands; Department of Internal Medicine, Division of Nephrology, University of Groningen, University Medical Center Groningen, Groningen, The Netherlands; Department of Pathology and Medical Biology, University of Groningen, University Medical Center Groningen, Groningen, The Netherlands

**Keywords:** Vasculitis, GPA, Cytokines, B-cells, Relapse, IL10, TNFα, CCL19, sCD27

## Abstract

**Background:**

B cells are capable of producing regulatory and effector cytokines. In patients with granulomatosis with polyangiitis (GPA), skewing of the pro- and anti-inflammatory cytokine balance may affect the risk of relapse. This study aimed to investigate differences in B cell cytokine production in patients with relapsing GPA and in controls, and determine whether this can aid in relapse prediction.

**Methods:**

Thirteen GPA patients with an upcoming relapse were matched with non-relapsing patients and healthy controls in a retrospective design. The B cell subset distribution was determined from peripheral blood. Cryopreserved peripheral blood mononuclear cells were cultured and intracellular B cell production of regulatory (IL10) and effector (TNFα, IFNγ, IL2, IL6) cytokines was assessed. Finally, serum markers associated with B cell activation (sCD27) and migration (CCL19) were determined.

**Results:**

GPA patient samples exhibited significantly lower percentages of TNFα+ B cells than controls, an effect that was most pronounced in patients about to relapse. B cell capacity for IL10 production was similar in patients and controls. No significant differences were observed for cytokine production in relapsing and non-relapsing GPA patients. TNFα production correlated strongly with IL2, IFNγ and the percentage of memory B cells. No change in effector cytokines occurred before relapse, while the percentage of IL10+ B cells significantly decreased. GPA patients in remission had increased serum levels of CCL19 and sCD27, and sCD27 levels increased upon active disease.

**Conclusions:**

While differences in effector B cell cytokine production were observed between patients and controls, monitoring this in GPA did not clearly distinguish patients about to relapse. Prospective measurements of the regulatory cytokine IL10 may have potential for relapse prediction. Memory B cells appear mainly responsible for effector cytokine production. Increased migration of these cells could explain the decreased presence of TNFα+ B cells in the circulation.

**Electronic supplementary material:**

The online version of this article (doi:10.1186/s13075-016-0978-1) contains supplementary material, which is available to authorized users.

## Background

Anti-neutrophil cytoplasmic antibodies (ANCA) are associated with chronic inflammatory small vessel vasculitides such as granulomatosis with polyangiitis (GPA). Patients with GPA frequently have circulating autoantibodies directed against the neutrophil constituent proteinase-3 (PR3) [1]. GPA is a relapsing disorder and while numerous risk factors for relapse have been described, no good markers are available to predict upcoming relapses in individual patients [2, 3]. B cells are important effector cells in autoimmune disease pathogenesis, not only as the producers of autoantibodies, but also as antigen presenting cells and cytokine producers [4]. B cell depletion therapy using the anti-CD20 monoclonal antibody rituximab has proven to be an effective therapeutic strategy for inducing remission in GPA [5, 6], indicating a pathogenic role for these cells. Clinical improvement in rituximab-treated patients can precede the reduction in autoantibody titers [7], highlighting the importance of antibody-independent mechanisms of B cells. Moreover, a subset of B cells has been ascribed with regulatory functions through the production of anti-inflammatory cytokines like interleukin (IL)10 [8]. These cells have been termed regulatory B cells (B_reg_) but there is no commonly accepted phenotypical description for this subset. It has been proposed that B_regs_ could be identified in the circulation by their surface markers, including CD24^high^CD38^high^ [9], CD24^high^CD27+ [10] and CD5+ B cells [11], although B_regs_ are functionally defined as IL10-producing B cells. Several studies have examined IL10 production in patients with ANCA-associated vasculitis (AAV). The results are inconclusive, as in one study patients with active AAV had lower production of IL10 [12], in another there was decreased IL10 production in patients with active disease and in patients in remission [13], while in a third study there were no differences compared to healthy controls [14]. None of these studies examined whether IL10 production changes in individual patients prior to relapse, or investigated production of other cytokines. This may be relevant because B cells are also capable of producing proinflammatory cytokines [15].

For these effector B cells (B_eff_) numerous effects on the immune response have been described in mouse models [16]. Tumor necrosis factor (TNF) expressed by B cells promotes T-helper 1 (Th1) differentiation, leading to amplification of interferon (IFN)γ production by CD4+ and CD8+ T cells [17]. IFNγ produced by B cells could also support Th1 responses [18], and promote macrophage activation [19]. B cells can promote Th2 memory responses through production of IL2 [20] and produce large quantities of IL6, shown capable of increasing disease pathogenesis in experimental autoimmune encephalomyelitis models through activation of Th17 cells [7]. Moreover, several cytokines, including IL6 and TNFα, can support the survival of plasma cells [21]. Collectively, these observations indicate that cytokine production by B cells might be an important factor in autoimmune disease pathogenesis. In patients with GPA the B_reg_ and B_eff_ cytokine production may be a factor that affects the balance between remission and relapse. However, data on human B cell cytokine production in autoimmunity are scarce. In the present study we investigated whether production of proinflammatory and anti-inflammatory cytokines by B cells in patients with GPA deviates from that in healthy individuals and examined whether analysis of the B cell cytokine production profile could help predict upcoming relapses. As B cell cytokine production from peripheral cells may be affected by B cell migration and activation, markers for these processes were also assessed.

## Methods

### Study population

A cohort of 84 patients with PR3-ANCA-positive GPA was prospectively monitored for 15–24 months. Clinical parameters, including current therapy, were recorded and peripheral blood mononuclear cells (PBMC) and serum samples were stored. Sixteen patients relapsed during the period of sample collection. Relapses were based on clinical judgement and had to result in the decision to increase or initiate immunosuppressive therapy. From the patients who went on to have a future relapse, a retrospective selection was made based on availability of PBMC and the presence of more than 3 % of B cells within the lymphocyte population. This resulted in a selection of 13 patients, who were individually age- and sex-matched with 13 patients with GPA, who did not relapse for at least 1.5 years and 13 healthy controls, prior to data analysis. The median time between sampling and relapse was 74 (range 14–157) days. Samples from an earlier time point in remission were also available for 11 patients who relapsed, and the median time between the two samples prior to relapse was 98 (range 38–140) days. The diagnosis of GPA was based on definitions outlined in the Chapel Hill Consensus Conference [22] and all patients fulfilled the classification criteria of the American College of Rheumatology [23]. All subjects gave written informed consent according to the Declaration of Helsinki and the study was approved by the Medical Ethical Committee of the University Medical Center Groningen (METc UMCG 2012/151). Patient and control characteristics are listed in Table 1.

**Table 1:** Characteristics of patients with GPA and healthy controls

	HC	GPA, no future relapse	GPA, future relapse
Subjects, n (% male)	13 (46)	13 (46)	13 (46)
Age, years, mean (range)	55 (44–74)	52 (32–76)	52 (32–75)
PR3-ANCA titer, median (range)		1:40 (0 to >640)	1:80 (20 to >640)
Disease duration, years, median (range)		12.6 (5.5–31.5)	15.3 (2.3–24.3)
Number of previous relapses, median (range)		2 (0–5)	5 (1–10)
Time to relapse after sample, days, median (range)		n/a	74 (14–157)
Disease form, n (%)			
Localized		0 (0)	0 (0)
Early systemic		2 (15.4)	2 (15.4)
Generalized		8 (61.5)	9 (69.2)
Severe		3 (23.1)	2 (15.4)
Treatment at time of sampling, n (%)			
Aza		1 (7.7)	3 (23.1)
Pred		1 (7.7)	1 (7.7)
MMF + pred		2 (15.4)	3 (23.1)
No immunosuppressive therapy		9 (69.2)	6 (46.2)
B cell phenotype in %, median (range)			
Total CD19+ B cells	12.3 (6.6–22.24)	9.8 (6.3–28.6)	9.9 (3.4–19.9)
Transitional B cells	10.77 (3.9–17.6)	9.2 (4.8–18.1)	9.3 (3.1–11.7)
Naive B cells	63.6 (37.4–83.4)	79.0 (74.5–90.1)	79.3 (60.1–85.7)
Memory B cells	22.4 (6.6–54.0)	8.1 (4.2–29.7)	8.2 (2.3–19.5)
CD24^high^CD38^high^ B cells	6.6 (2.2–9.6)	4.6 (2.1–11.8)	5.3 (0.9–8.5)
CD24^high^CD27+ B cells	15.5 (3.5–45.4)	3.3 (2.1–24.8)	3.4 (0.7–10.8)
CD5+ B cells	23.4 (12.1–57.6)	19.8 (12.1–64.2)	18.9 (4.7–49.9)

*ANCA* anti-neutrophil cytoplasmic antibody, *Aza* azathioprine, *GPA* granulomatosis with polyangiitis, *HC* healthy controls, *MMF* mycophenolate mofetil, *n/a* not applicable, *PR3* proteinase-3, *Pred* prednisolone

Clinical data of the individual patients at time of relapse is listed in Additional file 1 : Table S1.

### Flow cytometry for analysis of the B cell phenotype

Blood was collected in EDTA tubes, and 100 μl was incubated with anti-human CD19-eFluor450 (eBioscience, San Diego, CA, USA), CD24-fluorescein isothiocyanate (FITC) (BD biosciences, Franklin Lakes, NJ, USA), CD27-APC-eFluor780 (eBioscience), CD38-PeCy5 (eBioscience), CD5-PerCp-Cy5.5 (Biolegend, San Diego, CA, USA) or the corresponding isotype controls. After 15 minutes cells were treated with fluorescence-activated cell sorting (FACS) Lysing solution (BD Biosciences). Samples were measured using an LSR-II flow cytometer (BD biosciences) and data were analyzed using Kaluza 1.2 flow analysis software (Beckman Coulter, Brea, CA, USA). B cells were divided into transitional, memory, naive, CD24^high^CD38^high^ and CD24^high^CD27+ B cells as described previously [14]. CD5+ B cells were gated on an isotype control.

### Cell culture and intracellular B cell cytokine pattern upon in vitro stimulation

PBMC were isolated and stored in RPMI 1640 (Lonzo, Basel, Switzerland) supplemented with 50 μg/mL gentamycin (GIBCO, Life Technologies, Grand Island, NY, USA), 10 % fetal calf serum (FCS) (Lonza) and 10 % dimethyl sulfoxide. The cryopreserved PBMC were thawed, concentrations were adjusted to 10^6^ cells/mL in RPMI + 10 % FCS, and cells were seeded in 24-well flat bottom plates (Corning, NY, USA). Cells were left untreated or stimulated using 500 ng/mL CpG-oligodeoxynucleotides (ODN) 2006 (Hycult Biotech, Uden, the Netherlands). Culture plates were incubated for 72 h at 37 °C with 5 % CO2. During the last 5 h of incubation 50 ng/mL phorbol myristate acetate (PMA; Sigma-Aldrich, St Louis, MO, USA), 2 mM calcium ionophore (CaI; Sigma-Aldrich) and/or 10 μg/mL Brefeldin A (BFA; Sigma-Aldrich) were added to the cell culture. Cells were harvested and stained using anti-human CD19-eFluor450 and CD22-PeCy5 (Biolegend). Subsequently cells were fixed and permeabilized for intracellular staining using a Fix&Perm kit (Invitrogen, Life Technologies, Grand Island, NY, USA) and incubated with antibodies against human IL10-PE (Biolegend), TNFα-Alexa Fluor 488 (BD biosciences), IL6-APC (eBioscience), IL2-PeCy7 (eBioscience) and IFNγ-Alexa Fluor 700 (BD biosciences). Samples were measured with an LSR-II flow cytometer and data were analyzed using Kaluza 1.2. Samples that had not been stimulated with PMA and CaI were used as negative controls to set the gates during data analysis. Data are presented as the percentage of cytokine-positive B cells within the total CD19+CD22+ population.

### Enzyme-linked immunosorbent assay (ELISA) for CCL19 and soluble CD27

Serum samples from healthy controls and patients had been collected and stored at −80 °C on the same day as PBMC storage and B cell phenotype analysis. Moreover, serum samples from the relapsing patients were available from the time of active disease. A Human CCL19/MIP-3 beta DuoSet ELISA (R&D Systems, Minneapolis, MN, USA) and a PeliKine Compact™ human soluble CD27 ELISA (Sanquin, Amsterdam, the Netherlands) were performed according to the manufacturers’ instructions. CCL19 levels are expressed as pg/mL and sCD27 levels as units (U)/mL.

### Statistical analysis

Statistical analysis was performed using SPSS v22 (IBM Corporation, Chicago, IL, USA) and GraphPad Prism v5.0 (GraphPad Software, San Diego, CA, USA). Data are presented as median values with the interquartile range, unless stated otherwise. For comparison between groups the unpaired *t* test was applied for data with a Gaussian distribution and the Mann-Whitney *U* test was used for data with a non-Gaussian distribution. For intra-individual comparisons the paired *t* test or Wilcoxon matched pairs test was performed for Gaussian- and non-Gaussian-distributed data, respectively. Correlation analysis was performed by calculating the Spearman rank correlation coefficient. *P* values <0.05 were considered statistically significant.

## Results

### B cell subset distribution in patients with GPA and healthy controls

The B cell subset distribution in patients with GPA in remission differed from healthy controls (HC). Specifically, patients with GPA had lower percentages of CD27+ memory B cells (*p* = 0.0014), CD24^high^CD27+ B cells (*p* < 0.001) and higher percentages of naive B cells (*p* < 0.001) than healthy controls (Table 1). These same differences were observed when patients who were on immunosuppressive treatment at that time were excluded from analysis. No significant differences were found when directly comparing patients who did or did not have a future relapse.

### Intracellular B cell cytokine pattern in patients with GPA and healthy controls

**Fig. 1:**
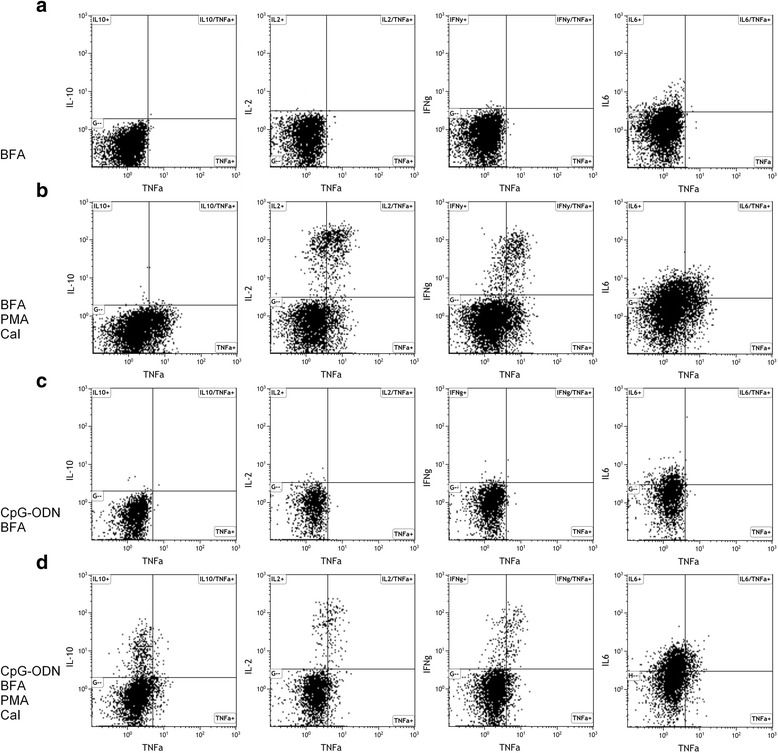
Flow cytometry gating example. Representative flow cytometry dot plots of CD19+CD22+ B cells. **a** Brefeldin A (*BFA*)-treated samples. **b** Phorbol myristate acetate (*PMA*)- and calcium ionophore (*CaI*)-stimulated, BFA-treated samples. **c** CpG-oligodeoxynucleotide (CpG-ODN)-stimulated, BFA-treated samples. **d** CpG-ODN-, PMA-, and CaI-stimulated, BFA-treated samples. To determine the percentage of B cells positive for cytokine production (**a**) and (**c**) were used as negative controls to set the gates for (**b**) and (**d**), respectively

PBMC were cultured with or without CpG for 3 days, followed by PMA and CaI stimulation for 5 h. In all samples the production of TNFα, IL2, IFNγ, IL6 and IL10 was determined by flow cytometry (Fig. 1). IL10 production was only clearly detectable when samples had been stimulated with CpG; without CpG IL10 was detected in less than 0.5 % of B cells. In contrast, for the effector cytokines there were clear positive populations both with and without CpG stimulation. Simultaneous production of regulatory and effector cytokines was also observed in B cells. For example, the median double positivity for IL10 and TNFα was 20 % of the total IL10-positive B cell population; this proportion was similar for patients with GPA and controls. Moreover, the B_eff_ cytokines IL2, IFNγ and TNFα were regularly produced together by B cells.

First, all GPA patients in remission were compared to the healthy controls. In samples not incubated with CpG a lower percentage of TNFα-producing B cells was present in samples from patients with GPA (median 7.3 %, interquartile range 5.5–10.0 %) compared to healthy controls (12.3, 8.7–18.8 %, *p* = 0.003). Expression of the other cytokines was not significantly different between controls and patients, although numerically the percentages of B cells positive for IL2 (6.7, 4.7–8.2 % in HC vs 5.1, 2.7–6.8 % in GPA) and IFNγ (6.0, 3.8–6.7 % vs 4.2, 3.3–4.9 %) were lower in patients, while IL6 (21.2, 15.6–26.3 % vs 26.2, 20.9–31.1 %) appeared mildly increased in GPA. In samples stimulated with CpG no significant differences were observed for B cell production of IL10 between healthy controls (6.7, 3.8–9.7 %) and patients with GPA (6.5, 3.6–9.6 %), or for the effector cytokines (data not shown). Stimulation with CpG also affected the production of IL6, by inducing a significant increase in IL6+ B cells in both patient and control samples *(p* = 0.0014*)*. Furthermore, a significant decrease in TNFα+ B cells was seen upon CpG stimulation in healthy control samples (*p* = 0.017), whereas in patients there was a more diverse response. The results for samples not stimulated with CpG were used for the remaining analyses, with the exception of IL10.

### Changes in B cell cytokine production before relapse

**Fig. 2:**
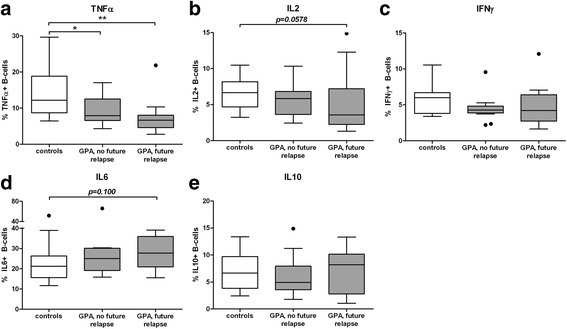
B cell cytokine production in (relapsing) patients with granulomatosis with polyangiitis (*GPA*) and in controls. Peripheral blood mononuclear cells were cultured without CpG-oligodeoxynucleotides (CpG-ODN) (**a**-**d**) and with CpG-ODN (**e**). Total percentages of cytokine-producing B cells were determined within the CD19+CD22+ B cell population. Patients were divided into those who relapsed soon after the sampling (*future relapse*) and those that did not relapse for at least 1.5 years (*no future relapse*). *Graphs* represent data from 13 relapsing patients, 13 non-relapsing patients and 13 healthy controls. In the *box and whisker plots* (Tukey), *boxes* represent median values and the interquartile range: *p < 0.05, **p < 0.01

The patients with GPA in remission were divided into those who were diagnosed with a relapse after sampling and those who had not relapsed for at least 1.5 years after sampling. These groups were compared with each other and with the HC. There were no significant differences in production of the regulatory cytokine IL10 between the patient groups and the HC. The differences we observed in the effector cytokine production for patients with GPA and controls were most notable in the patients who had relapsed after sampling (Fig. 2). There were fewer TNFα+ B cells in the samples from non-relapsing patients (7.9, 5.5–10.0 %, *p* = 0.045) compared to the control samples, but when comparing relapsing patients (6.7, 4.6–8.0 %, *p* = 0.002) to controls this difference appeared to be more pronounced. Relapsing patients also appeared to have fewer IL2+ B cells than controls, albeit not significantly so (3.6, 2.3–7.2 %, *p* = 0.058). This was the same for the increased proportion of IL6+ B cells in relapsing patients (27.7, 20.9–35.9 %, *p* = 0.10) compared to HC. However, when compared directly, there was no significant difference in the production of any cytokine in the relapsing and non-relapsing patients.

**Fig. 3:**
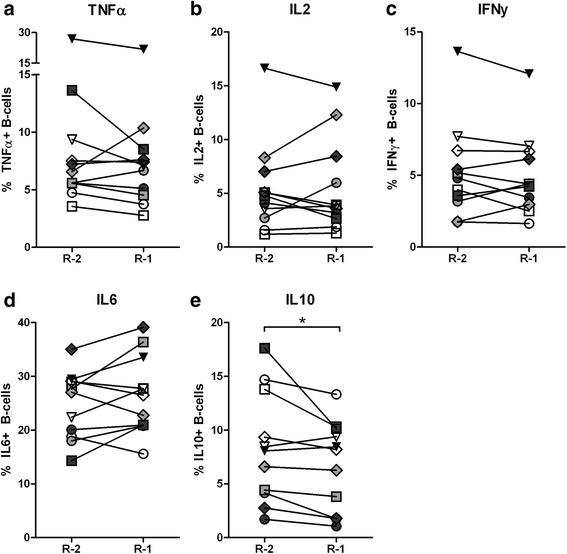
Changes in B cell cytokine production before relapse. For 11 relapsing patients two consecutive time points before relapse were analyzed simultaneously, to determine whether changes in cytokine production occurred prior to relapse. Peripheral blood mononuclear cells were cultured without CpG-oligodeoxynucleotides (CpG-ODN) (**a**-**d**) and with CpG-ODN (**e**). Total percentages of cytokine-producing B cells were determined within the CD19+CD22+ B cell population. *Graphs* represent data from 11 patients and each *connected line* represents an individual patient. The different *symbols* indicate the individual patients in each subgraph. *R-1* indicates the sample taken closest to relapse and *R-2* the sample taken before. *P < 0.05

Two consecutive samples before relapse were available for 11 patients, and changes in cytokine production were analyzed. No clear change in the production of effector cytokines occurred prior to relapse (Fig. 3a-d), while there was a significant decrease in IL10+ B cells (Fig. 3e). In eight patients in sustained remission, no significant change was observed in the production of IL10 between two consecutive samples (Additional file 1: Figure S1).

**Fig. 4:**
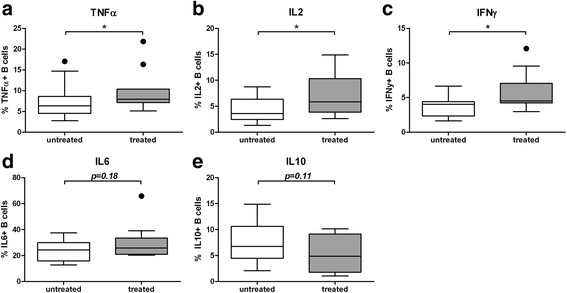
Current medication use and B cell cytokine production. **a-e** All patients with granulomatosis with polyangiitis (GPA) were divided into groups based on whether they were receiving immunosuppressive treatment at the time of sampling. *Graphs* represent data for 11 patients who were treated at the time of sampling and 15 who were not treated. In the *box and whisker plots* (Tukey), the *boxes* represent median values and the interquartile range. *P < 0.05

As there was a discrepancy in the proportion of treated patients in the relapse and non-relapse groups (albeit not significant) we compared all patients who were currently on immunosuppressive treatment (n = 11) to those who were not (n = 15). The percentages of TNFα-, IL2-, and IFNγ-producing cells were significantly higher in patients who were currently being treated (Fig. 4a-c); as more patients were treated in the relapse group this is unlikely to have affected the significance of our results. IL10 and IL6 production was not significantly different in treated and untreated patients (Fig. 4d-e).

### Correlation analysis for the B cell phenotype and cytokine production

The results from the correlation analysis for the B cell subset distribution and B cell cytokine production are summarized in Table 2. Results are from the combined cohorts of patients with GPA (n = 26) and healthy controls (n = 13). There was strong correlation between the effector cytokines TNFα, IL2 and IFNγ. Production of these cytokines, in particular TNFα, also demonstrated a strong positive correlation with the percentage of circulating CD27+ memory B cells. These results were also observed when patients 10.1186/s13075-016-0978-1 correlation with the percentage of circulating CD27+ memory B cells. These results were also observed when patients with GPA were analyzed separately. There was positive correlation with the production of IL6 and IL10 in CpG-stimulated samples. There was no correlation between these cytokines and any specific B cell subset. Interestingly, there was no correlation between the production of IL10 and any of the proposed B_reg_ populations.

**Table 2:** Correlation analysis results

		TNFα+, %	IL6+, %	IL2+, %	IFNγ+, %	IL10+, %
Memory, %	Spearman’s rho	0.864*	-0.063	0.586*	0.576*	0.053
	*P* value	<0.0001*	0.702	<0.0001*	0.0001*	0.747
CD24^high^CD38^high^, %	Spearman’s rho	-0.237	-0.292	-0.364*	-0.168	0.049
	*P* value	0.146	0.071	0.023*	0.308	0.768
CD24^high^CD27+, %	Spearman’s rho	0.834*	-0.115	0.553*	0.467*	0.043
	*P* value	<0.0001*	0.487	<0.001*	0.003*	0.797
CD5+, %	Spearman’s rho	-0.199	0.021	-0.132	-0.214	0.047
	*P* value	0.291	0.914	0.488	0.257	0.804
TNFα+, %	Spearman’s rho		-0.012	0.733*	0.663*	0.237
	*P* value		0.940	<0.0001*	<0.0001*	0.146
IL6+, %	Spearman’s rho			0.100	0.124	0.364*
	*P* value			0.545	0.451	0.023*
IL2+, %	Spearman’s rho				0.527*	-0.061
	*P* value				0.001*	0.714
IFNγ+, %	Spearman’s rho					-0.085
	*P* value					0.607

*IFN* interferon, *IL* interleukin, *TNF* tumor necrosis factor. *Statistically significant results

### CCL19 and sCD27 serum levels are increased in patients with GPA

**Fig. 5:**
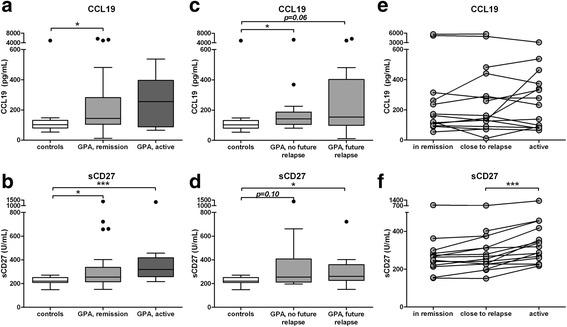
Serum CCL19 and sCD27 levels in (relapsing) patients with granulomatosis with polyangiitis (*GPA*) and controls. Serum levels of CCL19 and sCD27 were determined by ELISA. **a**, **b** Patients in remission and with active disease were compared to healthy controls. **c**, **d** Patients in remission were divided into those who relapsed soon after the sampling (*future relapse*) and those that did not relapse for at least 1.5 years (*no future relapse*). In the *box and whisker plots* (Tukey), *boxes* represent the median values and interquartile range. **e**, **f** The relapsing patients were compared for two consecutive time points in remission and the ensuing active disease time point. Each *line* represents an individual patient. *P < 0.05, ***p < 0.001

As results were obtained using peripheral blood cells, differences in B cell migration and activation were investigated by analyzing the B cell trafficking chemokine CCL19 [24, 25] and soluble CD27 serum levels, using ELISA. CCL19 was significantly increased in patients with GPA in remission (144, 105–281 pg/mL) compared to healthy controls (102, 81–131; Fig. 5a). When patients in remission were divided based on future relapse, no significant difference was observed in CCL19 serum levels between patients who did (153, 99–402) and did not (142, 105–187) relapse after sampling (Fig. 5c). CCL19 levels in relapsing patients did not change between the two time points before the relapse, or upon active disease (Fig. 5e). There was no correlation between serum levels of CCL19 and the B cell phenotype distribution.

Serum levels of sCD27 were significantly increased in patients in remission (255, 216–337 U/mL) and patients with active disease (319, 259–418) compared to healthy controls (221, 210–251; Fig. 5b). Concentrations were not different when patients were divided into those with (261, 227–360) and without (255, 213–407) an upcoming relapse (Fig. 5d). While no clear change in sCD27 levels occurred prior to relapse, when patients presented with active disease, there was a significant increase in sCD27 (median 21 %) (Fig. 5f). There was no correlation between sCD27 serum levels and the percentage of circulating CD27+ memory cells, or other B cell subsets in remission patients or controls.

## Discussion

The antibody-independent functions of B cells are of increasing interest. This includes the production of both proinflammatory and anti-inflammatory cytokines. In particular, the production of IL10 and the presence of B_regs_ in autoimmune disease are currently under investigation. Scant information is available about B cell cytokine production in patients with GPA, especially B_eff_ cytokine production, and how this may relate to an upcoming relapse. Here we describe the B cell cytokine production profile in patients with GPA in remission, patients about to relapse, and matched healthy controls.

The most prominent result of this analysis is that patients with GPA had a significantly lower percentage of TNFα-producing B cells in the circulation, and this was especially notable in patients who were about to relapse. TNFα-producing B cells correlated very strongly with the presence of CD27+ memory B cells in the circulation. We observed that patients with GPA had a decreased proportion of CD27+ memory B cells in peripheral blood, confirming our previously described results [14]. Moreover, we have found that CD27+ memory B cells are even further decreased in patients prior to relapse (unpublished data). CD27+ memory B cells have a greater capacity to produce TNFα than naive B cells in patients with rheumatoid arthritis (RA) [26]. It is likely that the reduced proportion of CD27+ memory cells in patients with GPA [14] explains the lower percentage of TNFα-producing cells in our study. Both IL2 and IFNγ production correlated strongly with the production of TNFα and the percentage of CD27+ memory B cells, suggesting that effector B cells produce several proinflammatory cytokines simultaneously.

One potential explanation for the decreased presence of TNFα-producing CD27+ memory B cells in the circulation is a higher rate of B cell trafficking in GPA, which mainly affects the memory B cells. As a first attempt to investigate this option the B cell trafficking chemokine CCL19 was measured in serum samples. CCL19 has been described to especially enhance the migration of CD27+ memory B cells [25]. Levels of CCL19 were indeed increased in patients with GPA compared to controls, suggesting that B cell migration is intensified in these patients. In patients with RA the level of serum CCL19 has been weakly and inversely correlated with the frequency of circulating CD27+ memory B cells [27] and increased levels of CCL19 have been found in synovial tissue [28]. Moreover, CCL19 is involved in the formation of ectopic lymphoid structures [24]. We did not observe correlation between CCL19 and the percentage of CD27+ cells in the GPA cohort or in the healthy controls. However, for the correlation reported in RA the correlation coefficient was not high, and the number of subjects in our study is substantially lower. Increased migration of CD27+ memory B cells is a viable option to explain their decreased proportion in the circulation of patients with GPA. Investigation of tissue biopsies is warranted to prove that. Concentrations of CCL19 did not change before or at the time of relapse, indicating that CCL19 cannot differentiate between patients with active disease and those in remission, and it is not a useful marker to predict relapses.

Soluble CD27 is a marker for both B cell and T cell activation [29, 30]. Levels of sCD27 were significantly increased in GPA compared to controls. In contrast to CCL19, the concentration of serum sCD27 significantly increased with active disease. The increased sCD27 may be a reflection of lymphocyte activation in GPA, which increases upon relapse. While not predictive for disease relapse, sCD27 could potentially be a marker for active disease. Another explanation for increased sCD27 is increased shedding of the receptor. Surface CD27 can be cleaved through the action of matrix metalloproteinases (MMPs) [31]. As patients with active GPA have upregulation of several MMPs [32] this could explain the increased sCD27 in samples from patients with active disease. Moreover, the presence of CD27-negative memory B cells has been described and it has been speculated that the CD27-negative memory compartment contains autoreactive B cells that have downregulated their activation molecules in an attempt to limit autoreactivity [33]. While these cells are scarce in healthy individuals, they are significantly increased in patients with systemic lupus erythematosus (SLE) [34], and serum levels of sCD27 are also increased in SLE patients compared to controls [35]. The CD27-negative B cells could be a source of sCD27 present in the serum; however, as T cells can also express CD27 it is not the only possible source.

With regard to the B_reg_ cytokine IL10, there were no differences between controls and (relapsing) patients in the total percentages of IL10-producing B cells. This result is in line with our previous observations, which showed a similar capacity for IL10 production by B cells in AAV patients and controls [14]. However, there was a significant decrease in IL10 production in individual patients approaching a relapse, which was not seen in patients in remission, indicating that B cell regulatory capacity may decrease in individual patients prior to relapse. Further investigation is warranted to fully confirm these changes.

The production of IL10 did not correlate with any of the proposed surface marker phenotypes for B_regs_. However, none of these phenotypes have been fully confirmed as clear B_reg_ populations. Moreover, the stimulation with CpG for 3 days may affect the phenotype of B cells in culture, resulting in a different subset distribution. For one, CpG has been reported to induce naive B cell proliferation without causing maturation into memory cells [36], and it can prolong the lifespan of mature-naive B cells in vitro [37]. Such changes could help explain the lack of correlation between a phenotype analysis in the peripheral blood and cytokine expression after CpG stimulation, although one study has shown that 66 h stimulation with CpG does not significantly affect the B cell subset distribution [38]. Regardless, additional phenotypical analysis post CpG stimulation may give a clearer picture of which B cells are responsible for the production of IL10 in this setting.

IL10 production did correlate with the production of IL6 in Toll-like receptor (TLR)9-activated B cells, even though IL6 is generally regarded as a proinflammatory cytokine, and its overproduction has been related to the onset of autoimmunity [39]. Moreover, blockade of the IL6 receptor has proven to be an effective therapeutic target in several autoimmune diseases, including RA [40] and giant cell arteritis [41]. Marginal zone B cells that produced high levels of IL6 in response to TLR4 stimulation simultaneously produced increased levels of IL10 [7]. These cytokines can be separated, for example, calcineurin antagonists have been demonstrated to selectively inhibit IL10 release without affecting secretion of IL6 in TLR9-stimulated B cells [42]. In addition, we demonstrated here that B cells can produce IL10 and TNFα concomitantly, which has been described previously, with CD24^high^CD27+ B cells having the largest fraction of TNFα/IL10 dual positivity [43]. The capacity of certain B cells to demonstrate both regulator and effector roles indicates that the in vivo context is crucial in determining the B cell function. While we demonstrated here that patients with GPA and healthy individuals have a similar capacity for B cell IL10 production after TLR9 activation, differences in the in vivo environment could result in different B cell cytokine production profiles.

There are limitations to our study, mainly due to the restricted number of subjects that could be included. Statistical analyses have not been corrected for multiple testing, and we lacked sufficient power to correct for multiple potential confounding factors. However, it was clear that current immunosuppressive treatment was not the underlying cause for the differences between patients and controls. Furthermore, total B cell cytokine production was determined here; it would be interesting to sort specific B cell subsets, for example, memory B cells, and determine cytokine production. Finally, results determined from peripheral samples may not be representative of active disease sites; however, biopsy studies will not lead to biomarkers that are easy to measure.

## Conclusions

Patients with GPA have a decreased percentage of TNFα-producing B cells compared to controls. This appears to be most prominent in those patients who are about to relapse. However, effector B cell cytokine production was not significantly different between relapsing and non-relapsing patients, and could not clearly differentiate which patients were about to relapse. IL10 production by B cells did decrease before relapse, and monitoring this may have potential for relapse prediction. Production of the effector cytokines TNFα, IL2, and IFNγ by B cells is highly correlated with the presence of CD27+ B memory cells in the circulation. Increased CCL19 levels suggest greater B cell migration in GPA, which may particularly affect memory B cells. The resulting changes in the B cell subset distribution in GPA are likely responsible for the aberrant proinflammatory cytokine production.

## Additional file

Additional file 1: Supplementary Table 1: Clinical data for individual patients and at moment of relapse. Supplementary Figure 1. IL10 in remission patients. Peripheral blood mononuclear cells were cultured CpG-ODN. Total percentages of cytokine producing B-cells were determined within the CD19+CD22+ B-cell population. Graph represents 8 patients in sustained remission, without immunosuppressive treatment. NRfirst indicates the earlier sample taken from the non-relapsing patients, NR-second the sample taken at a consecutive visit. (PDF 224 kb)

## Abbreviations

AAV: anti-neutrophil cytoplasmic antibody-associated vasculitis
ANCA: anti-neutrophil cytoplasmic antibodies
B_eff_: effector B cell
BFA: brefeldin A
B_reg_: regulatory B cell
CaI: calcium ionophore
CpG-ODN: CpG-oligodeoxynucleotides
ELISA: enzyme-linked immunosorbent assay
FCS: fetal calf serum
GPA: granulomatosis with polyangiitis
IFN: interferon
IL: interleukin
MMP: matrix metalloproteinase
PBMC: peripheral blood mononuclear cells
PMA: phorbol myristate acetate
PR3: proteinase-3
RA: rheumatoid arthritis
SLE: systemic lupus erythematosus
Th: T-helper
TLR: toll-like receptor
TNF: tumor necrosis factor
