# Supplementary material for: Regulatory and effector B cell cytokine production in patients with relapsing granulomatosis with polyangiitis
Source: Arthritis Res Ther. 2016 Apr 4;18:84. doi: 10.1186/s13075-016-0978-1 (PMC4820899; doi:10.1186/s13075-016-0978-1)
Supplement: Additional file 1: — Supplementary Table 1: Clinical data for individual patients and at moment of relapse. Supplementary Figure 1. IL10 in remission patients. Peripheral blood mononuclear cells were cultured CpG-ODN. Total percentages of cytokine producing B-cells were determined within the CD19+CD22+ B-cell population. Graph represents 8 patients in sustained remission, without immunosuppressive treatment. NRfirst indicates the earlier sample taken from the non-relapsing patients, NR-second the sample taken at a consecutive visit. (PDF 224 kb) [file 13075_2016_978_MOESM1_ESM.pdf]

## SUPPLEMENTARY DATA

### Supplementary Table 1: Clinical data for individual patients and at moment of relapse

| | | | Remission sample (used in this study) | | | | Time of relapse | | | | | |
|---|---|---|---|---|---|---|---|---|---|---|---|---|
| **Individual patients** | **M/F** | **age** | **CRP** | **Creatinine** | **ANCA titer** | **immunosuppressive treatment** | **Organ involvement** | **BVAS** | **CRP** | **Creatinine** | **ANCA titer** | **immunosuppressive treatment *** |
| R1 | F | 70 | 2,3 | 195 | > 640 | no | renal/joints | 13 | 110 | 270 | 320 | CYC/pred |
| R2 | F | 52 | 20 | 80 | 80 | low dose pred | ENT/lung | 10 | 10 | 68 | 80 | MMF/pred |
| R3 | M | 57 | <5 | 98 | 320 | MMF, low dose pred | renal/joints | 8 | 43 | 102 | > 640 | RTX/pred |
| R4 | F | 74 | 1,2 | 141 | > 640 | no | renal/joints | 15 | 9,3 | 186 | > 640 | CYC/pred |
| R5 | F | 32 | 9,3 | 62 | 80 | no | cardiac/joints | 9 | 147 | 66 | 160 | Pred/aza |
| R6 | M | 43 | 2,1 | 131 | 80 | low dose aza | ENT/eye/renal | 18 | 4,3 | 139 | 80 | Pred/RTX |
| R7 | F | 75 | 38 | 87 | 80 | Low dose MMF, low dose pred | lung | 5 | 51 | 94 | 160 | Pred/RTX/MMF |
| R8 | M | 53 | 2,5 | 77 | 40 | no | lung/ENT | 8 | 6,3 | 72 | 80 | Pred/MMF |
| R9 | M | 50 | 0,4 | 164 | 80 | no | ENT/joints/renal | 13 | 9,6 | 151 | 80 | Pred/RTX |
| R10 | F | 43 | 11 | 64 | 320 | aza | skin/renal | 9 | 18 | 67 | > 640 | Pred/CYC |
| R11 | M | 42 | 5,6 | 103 | 80 | no | ENT | 4 | 2,1 | 101 | 160 | Pred/aza |
| R12 | F | 35 | 13 | 77 | 20 | MMF, low dose pred | ENT/renal/lung | 19 | 150 | 73 | 20 | pred/RTX |
| R13 | M | 54 | 54 | 87 | 80 | aza | eye/joint | 3 | 11 | 79 | 40 | Pred/continuation of aza |
| | | | | | | | *median* | | *14* | *94* | *160* | |
| NR1 | F | 69 | 17 | 61 | 40 | no | | | | | | |
| NR2 | F | 52 | 0,8 | 84 | 0 | MMF, low dose pred | | | | | | |
| NR3 | M | 63 | 14 | 103 | 160 | MMF, low dose pred | | | | | | |
| NR4 | F | 76 | <5 | 142 | 40 | no | | | | | | |
| NR5 | F | 32 | 13 | 132 | 20 | no | | | | | | |
| NR6 | M | 42 | <5 | 90 | > 640 | no | | | | | | |
| NR7 | F | 73 | 1,8 | 81 | 20 | low dose pred | | | | | | |
| NR8 | M | 57 | 2,2 | 409 | 160 | no | | | | | | |
| NR9 | M | 52 | <5 | 81 | 40 | no | | | | | | |
| NR10 | F | 43 | 10 | 91 | 80 | no | | | | | | |
| NR11 | M | 43 | 2,8 | 97 | 80 | low dose aza | | | | | | |
| NR12 | F | 43 | 19 | 80 | > 640 | no | | | | | | |
| NR13 | M | 58 | 1,1 | 85 | 40 | no | | | | | | |
| *median* | | | *2.65* | *89* | *80* | | | | | | | |

Clinical data of patients that relapsed (R) after sampling, and those that did not (NR). R and NR patients were individually matched on age/sex. Relapses were based on clinical judgement and start or increase in immunosuppressives. BVAS and organ involvement at the moment of relapse are reported.
ANCA, anti neutrophil cytoplasmic antibody; Aza, azathioprine; BVAS, Birmingham Vasculitis Activity Score; CRP, C-reactive protein; CYC, cyclophosphamide; ENT, ear-nose-throat; MMF, mycophenolate mofetil; pred, prednisolone; RTX, rituximab
*Immunosuppressive treatment started after establishing the relapse. Prednisolone was increased to a dose between 0.5-1.0 mg/kg/day (mostly 1.0 mg/kg/day)

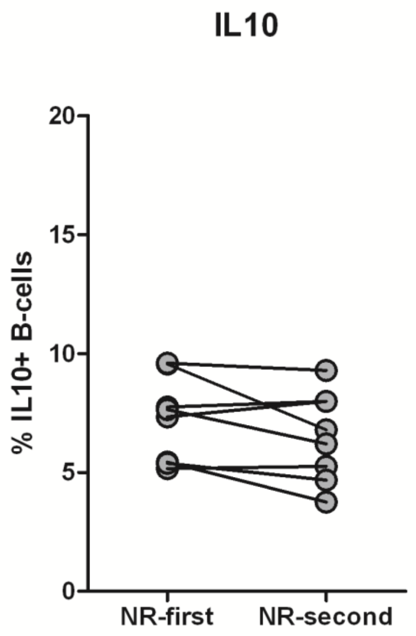


**Supplementary Figure 1. IL10 in remission patients.**
Peripheral blood mononuclear cells were cultured CpG-ODN. Total percentages of cytokine producing B-cells were determined within the CD19+CD22+ B-cell population. Graph represents 8 patients in sustained remission, without immunosuppressive treatment. NR-first indicates the earlier sample taken from the non-relapsing patients, NR-second the sample taken at a consecutive visit.
